# Supplementary material for: Magnitude of underweight, wasting and stunting among HIV positive children in East Africa: A systematic review and meta-analysis
Source: PLoS One. 2020 Sep 17;15(9):e0238403. doi: 10.1371/journal.pone.0238403 (PMC7498078; doi:10.1371/journal.pone.0238403)
Supplement: S1 Checklist — (DOC) [file pone.0238403.s001.doc]

| **Section/topic** | **#** | **Checklist item** | **Reported on page #** |
| --- | --- | --- | --- |
| **TITLE** | | |  |
| Title | 1 | Magnitude of underweight, wasting and stunting among HIV positive children in East Africa: a systematic review and meta-analysis | 1 |
| **ABSTRACT** | | |  |
| Structured summary | 2 | **Abstract**  **Background**: Malnutrition on the background of HIV infection is a complex medical condition that carries significant morbidity and mortality for affected children, with greater mortality from SAM among HIV-positive children than their HIV-negative peers. HIV-induced immune impairment heightened risk of opportunistic infection and can worsen nutritional status of children. HIV infection often leads to nutritional deficiencies through decreased food intake, mal-absorption and increased utilization and excretion of nutrients, which in turn can hasten death. Therefore, this systematic review and meta-analysis was aimed to estimate the pooled prevalence of underweight, wasting and stunting in the East African context.  **Methods:** Using PRISMA guideline, we systematically reviewed and meta-analyzed studies that examined the prevalence of underweight, wasting and stunting from PubMed, Cochrane Library, and Google Scholar. The data was extracted in excel sheet considering country, study design, year of publication prevalence reported. Then the authors transformed the data to STATA 14 for analysis. Heterogeneity across the studies was assessed by the Q and the I^2^ test. A weighted inverse variance random-effects model was used to estimate the magnitude of underweight, wasting and stunting. The subgroup analysis was done by country, year of publication, and study design. To examine publication bias, a funnel plot and Egger’s regression test were used.  **Results:** A total of 22 potential studies with 22074 participants were used for analysis. The pooled prevalence of under-weight, wasting, and stunting among HIV positive children in East Africa was found to be 41.63% (95%CI; 35.69–47.57; I^2^=98.7%; p<0.001), 24.65% (95%CI; 18.34–30.95; I^2^=99.2%; p<0.001), and 49.68% (95%CI; 42.59–56.77; I^2^=99.0%; p<0.001) respectively. The prevalence of under-weight among HIV positive children was found to be 49.67% in Ethiopia followed by 42.00 in Rwanda. It was high among cohort studies (44.87%). Based on the year of publication, the prevalence of under-weight among HIV positive children was found to be 40.88% from studies conducted from January 2008-December 2014, while it was 43.68% from studies conducted from 2015-2019. The prevalence of wasting among HIV positive children was found to be 29.7% in Tanzania followed by 24.94% in Ethiopia. Based on the study design, the prevalence of wasting among HIV positive children was found to be high in cohort studies (31.15%). The prevalence of stunting among HIV positive children was found to be 51.63% in Ethiopia, followed by 48.21% in Uganda.  **Conclusions**: The results presented above provide evidence of a higher prevalence of under nutrition among HIV positive children in East Africa. Despite the country level variations of child under nutrition in East Africa, still it is high in all aspects compared to the studies from other parts of Africa. It is recommended that further systematic review and meta-analysis need to be conducted on magnitude of malnutrition among HIV positive children in Sub-Saharan Africa as a whole.  **Keywords:** underweight, wasting, stunting, HIV/AIDS, East Africa | 1 |
| **INTRODUCTION** | | |  |
| Rationale | 3 | In East Africa, variety of previous studies have reported that the magnitude of under nutrition; accordingly the prevalence of under-weight, wasting and stunting was ranged from 19.4% to 77.1%, 7% to 77.1% and 13% to 71.8% respectively. This showed pronounced discrepancies among reports of under nutrition across different geographical settings and different time periods. Moreover, there is no regionally represented pooled data of under nutrition in East Africa. Therefore, this systematic review and meta-analysis was aimed to estimate the pooled prevalence of underweight, wasting and stunting in the East African context. | 4 |
| Objectives | 4 | To assess the pooled prevalence of underweight, wasting and stunting in the East African context | 4 |
| **METHODS** | | |  |
| Protocol and registration | 5 | This protocol follows the recommendations established by the Preferred Reporting Items for Systematic Reviews and Meta-Analyses (PRISMA) statement | 4 |
| Eligibility criteria | 6 | Inclusion and exclusion criteria In this systematic review and meta-analysis we have included cross-sectional, and cohort studies. Those studies had reported the prevalence of at least one under-weight, wasting and stunting and published in English language from January 2008 to December 2019. Studies conducted on marginalized groups/populations like children with any medical diseases, chronic diseases, or street mothers were excluded. Citations without abstract and/or full-text, anonymous reports, editorials, and qualitative studies were excluded from the analysis. The prevalence of under-weight, wasting, and stunting was considered when weight/ age, weight /height and height per age Z score <-2sd respectively within a specific population and multiply by 100 to be prevalence report. | 5 |
| Information sources | 7 | PubMed, Cochrane library, and Google Scholar were accessed. Articles with incomplete reported data were handled through contacting corresponding authors | 5 |
| Search | 8 | This review identified studies that provide data on the prevalence of under-weight, wasting, and stunting with the context of Eastern Africa. In the searching engine, mainly PubMed, Google Scholar, and Cochrane library were retrieved. The search included keywords that are the combinations of population, condition/outcome, and context. A snowball searching of the references of relevant papers for linked articles were also performed. Those search terms or phrases including were: “children”, “child”, “infant”, “under-nutrition”, “underweight”, “wasting”, stunting, and Eastern Africa. Using those key terms, the following search map was applied: (prevalence OR magnitude) AND (children [MeSH Terms] OR child OR infant) AND (under-nutrition [MeSH Terms] OR underweight OR wasting OR stunting) AND Eastern Africa on PubMed database (Table S1).Thus, the PubMed search combines #1 AND #2 AND #3 (Table S1). These search terms were further paired with names of each East African country. On both Cochran Library, and Google scholar, a build in text search were used on the advanced search section of the sources. | 4 and 5 |
| Study selection | 9 | Study selection and screening The retrieved studies were exported to Endnote version 8 reference managers to remove duplicate studies. Two investigators (BB and TG) independently screened the selected studies using article's title and abstracts before retrieval of full-text papers. We used pre-specified inclusion criteria to further screen the full-text articles. Disagreements were discussed during a consensus meeting with other reviewer (GT) for the final selection of studies to be included in the systematic review and meta-analysis.  In this systematic review and meta-analysis we have included cross-sectional, and cohort studies. Those studies had reported the prevalence of at least one under-weight, wasting and stunting and published in English language from January 2008 to December 2019. Studies conducted on marginalized groups/populations like children with any medical diseases, chronic diseases, or street mothers were excluded. Citations without abstract and/or full-text, anonymous reports, editorials, and qualitative studies were excluded from the analysis. The prevalence of under-weight, wasting, and stunting was considered when weight/ age, weight /height and height per age Z score <-2sd respectively within a specific population and multiply by 100 to be prevalence report. | 5 |
| Data collection process | 10 | Data extraction The authors developed data extraction form on the excel sheet in considering country, year of publication, study design and prevalence of underweight, wasting and stunting reported. The data extraction sheet was piloted using 4 papers randomly, and it was adjusted after piloted the template. Two of the authors extracted the data using the extraction form in collaboration. The third author checked the correctness of the data independently. Any disagreements between reviewers were resolved through discussions with a third reviewer when required. The mistyping of data was resolved through crosschecking with the included papers. | 6 |
| Data items | 11 | Underweight, wasting and stunting was considered when children have weight/age, wgt/hgt, and hgt/age less than -2 SD respectively | 6 |
| Risk of bias in individual studies | 12 | The pooled prevalence of under-weight, wasting, and stunting among HIV positive children in East Africa was found to be 41.63% (95%CI; 35.69–47.57; I^2^=98.7%; p<0.001), 24.65% (95%CI; 18.34–30.95; I^2^=99.2%; p<0.001), and 49.68% (95%CI; 42.59–56.77; I^2^=99.0%; p<0.001) respectively  We analyzed by random-effects model analysis and we did subgroup analysis. | 7 |
| Summary measures | 13 | We extracted and assessed the prevalence of underweight, wasting and stunting | 7 |
| Synthesis of results | 14 | Synthesis of results The authors transformed the data to STATA 14 for analysis after it was extracted in excel sheet. We pooled the overall prevalence estimates of underweight, wasting and stunting by a random effect meta-analysis model. We examined the heterogeneity of effect size using Q statistic and the I^2^ statistics. In this study, the I^2^statistic value of zero indicates true homogeneity, whereas the value 25, 50, and 75% represented low, moderate and high heterogeneity, respectively. Subgroup analysis was done by the study country, study design, and year of publication. Sensitivity analysis was employed to examine the effect of a single study on the overall estimation. Publication bias was checked by funnel plot and more objectively through Egger’s regression test. | 7 |

Page 1 of 2

| **Section/topic** | **#** | **Checklist item** | **Reported on page #** |
| --- | --- | --- | --- |
| Risk of bias across studies | 15 | The pooled prevalence of under-weight, wasting, and stunting among HIV positive children in East Africa was found to be 41.63% (95%CI; 35.69–47.57; I^2^=98.7%; p<0.001), 24.65% (95%CI; 18.34–30.95; I^2^=99.2%; p<0.001), and 49.68% (95%CI; 42.59–56.77; I^2^=99.0%; p<0.001) respectively  We analyzed by random-effects model analysis and we did subgroup analysis.  . | 7 |
| Additional analyses | 16 | The pooled prevalence of under-weight, wasting, and stunting among HIV positive children in East Africa was found to be 41.63% (95%CI; 35.69–47.57; I^2^=98.7%; p<0.001), 24.65% (95%CI; 18.34–30.95; I^2^=99.2%; p<0.001), and 49.68% (95%CI; 42.59–56.77; I^2^=99.0%; p<0.001) respectively. The prevalence of under-weight among HIV positive children was found to be 49.67% in Ethiopia followed by 42.00 in Rwanda. It was high among cohort studies (44.87%). Based on the year of publication, the prevalence of under-weight among HIV positive children was found to be 40.88% from studies conducted from January 2008-December 2014, while it was 43.68% from studies conducted from 2015-2019. The prevalence of wasting among HIV positive children was found to be 29.7% in Tanzania followed by 24.94% in Ethiopia. Based on the study design, the prevalence of wasting among HIV positive children was found to be high in cohort studies (31.15%). The prevalence of stunting among HIV positive children was found to be 51.63% in Ethiopia, followed by 48.21% in Uganda. | 7 |
| **RESULTS** | | |  |
| Study selection | 17 | A total of 3094 studies were identified; 2050 from PubMed, 12 from Cochrane Library, 1010 from Google Scholar and 22 from other sources. After duplication removed, a total of 970 articles remained (2127 removed by duplication). Finally, 230 studies were screened for full-text review, and 22 articles with (n=22074 patients) were selected for the analysis | 7 |
| Study characteristics | 18 | A total of 22 studies were included in this systematic review and meta-analysis . Of them 12 studies were done in Ethiopia , 1 in Kenya, while 2 were in Uganda, 1 in Rwanda , and 6 in Tanzania . Based on the study design used 14 studies were done by cross-sectional study design and while other 8 studies were conducted by cohort study design . 14/22(63.6%) were published between 2008 and 2014 and the remaining 8/22 (33.4%) were published between 2015 and 2019. The total number of participants in the included studies were ranges from 96 to 5951 | 8 |
| Risk of bias within studies | 19 | We have also checked publication bias and a funnel plot showed symmetrical distribution. | 13 |
| Results of individual studies | 20 | A total of 22 potential studies with 22074 participants were used for analysis. The pooled prevalence of under-weight, wasting, and stunting among HIV positive children in East Africa was found to be 41.63% (95%CI; 35.69–47.57; I^2^=98.7%; p<0.001), 24.65% (95%CI; 18.34–30.95; I^2^=99.2%; p<0.001), and 49.68% (95%CI; 42.59–56.77; I^2^=99.0%; p<0.001) respectively. The prevalence of under-weight among HIV positive children was found to be 49.67% in Ethiopia followed by 42.00 in Rwanda. It was high among cohort studies (44.87%). Based on the year of publication, the prevalence of under-weight among HIV positive children was found to be 40.88% from studies conducted from January 2008-December 2014, while it was 43.68% from studies conducted from 2015-2019. The prevalence of wasting among HIV positive children was found to be 29.7% in Tanzania followed by 24.94% in Ethiopia. Based on the study design, the prevalence of wasting among HIV positive children was found to be high in cohort studies (31.15%). The prevalence of stunting among HIV positive children was found to be 51.63% in Ethiopia, followed by 48.21% in Uganda. | 10 |
| Synthesis of results | 21 | The prevalence of under-weight among HIV positive children was found to be 49.67% in Ethiopia followed by 42.00 in Rwanda. It was high among cohort studies (44.87%). Based on the year of publication, the prevalence of under-weight among HIV positive children was found to be 40.88% from studies conducted from January 2008-December 2014, while it was 43.68% from studies conducted from 2015-2019. The prevalence of wasting among HIV positive children was found to be 29.7% in Tanzania followed by 24.94% in Ethiopia. Based on the study design, the prevalence of wasting among HIV positive children was found to be high in cohort studies (31.15%). The prevalence of stunting among HIV positive children was found to be 51.63% in Ethiopia, followed by 48.21% in Uganda. We analyzed by random-effects model analysis and we did subgroup analysis. Publication bias was checked by funnel plot and more objectively through Egger’s regression test . | 10 |
| Risk of bias across studies | 22 | The prevalence of under-weight among HIV positive children was found to be 49.67% in Ethiopia followed by 42.00 in Rwanda. It was high among cohort studies (44.87%). Based on the year of publication, the prevalence of under-weight among HIV positive children was found to be 40.88% from studies conducted from January 2008-December 2014, while it was 43.68% from studies conducted from 2015-2019. The prevalence of wasting among HIV positive children was found to be 29.7% in Tanzania followed by 24.94% in Ethiopia. Based on the study design, the prevalence of wasting among HIV positive children was found to be high in cohort studies (31.15%). The prevalence of stunting among HIV positive children was found to be 51.63% in Ethiopia, followed by 48.21% in Uganda. | 13 |
| Additional analysis | 23 | The prevalence of under-weight among HIV positive children was found to be 49.67% in Ethiopia followed by 42.00 in Rwanda. It was high among cohort studies (44.87%). Based on the year of publication, the prevalence of under-weight among HIV positive children was found to be 40.88% from studies conducted from January 2008-December 2014, while it was 43.68% from studies conducted from 2015-2019. The prevalence of wasting among HIV positive children was found to be 29.7% in Tanzania followed by 24.94% in Ethiopia. Based on the study design, the prevalence of wasting among HIV positive children was found to be high in cohort studies (31.15%). The prevalence of stunting among HIV positive children was found to be 51.63% in Ethiopia, followed by 48.21% in Uganda. | 19-21 |
| **DISCUSSION** | | |  |
| Summary of evidence | 24 | **Discussion**  Based on this review, it was found that the pooled prevalence of underweight in eastern Africa is 41%. This result is higher than the study conducted among HIV positive children, in Nigeria (12.1%), Cameroon (20.5%, 37.8%) and, and Burkina Faso (31%) respectively. But this result is lower compared to large scale study conducted in southern Africa(47.3%) **.** The discrepancy might be due to the difference in number of study participants across the studies.  The sub group analysis based on country revealed that, the pooled prevalence of under-weight among HIV positive children was found to be 49.67% in Ethiopia, followed by 42.00% in Rwanda. The result is higher compared to large scale DHS study in sub Saharan Africa, which accounts 31.2% in Ethiopia and 18.5% in Rwanda respectively , and EDHS 2109 mini report which accounts overall 21% . Based on the study design, the pooled prevalence of under-weight among HIV positive children was found to be 39.33% in cross-sectional studies and 44.87% in cohort studies respectively. This may be due to cohort studies apply strict follow-up trend of the patients; through this they can record more reliable reports of the patients overall character. The pooled prevalence of underweight on the studies conducted from (2015-2019) found to be increased (43.68%) compared to the studies conducted from January 2008-2014(40.88%). This indicates that underweight is still an alarming issue among HIV positive children’s in East Africa.  The pooled prevalence of wasting among HIV positive children in East Africa found to be 24.65% (95%CI; 18.34–30.95). This result is higher compared to the study conducted in central and West African countries (16%), the study conducted by **Pendal et al** in Cameroon(18.4%), and large scale study conducted in southern Africa(21.3%). The discrepancy might be due to the emphasis given by the government as well as stakeholders of the area regarding the effects of HIV/AIDS on child growth and development.  Regarding sub group analysis by country the prevalence of wasting is higher in Tanzania (29%) followed by Ethiopia (24.94%). This result is higher compared to the data reported by Ethiopian Demographic Health Survey (EDHS), 2019 mini report which accounted 7% of overall children wasted. This shows that these two countries needs great emphasis to decrease the burden of acute under-nutrition due to HIV infection; and it is better to invite governmental and non-governmental organizations regarding nutritional support to HIV-infected children at ART initiation which could help to decrease morbidity and mortality, and ensures an optimal growth and a better cognitive and social development. The prevalence of wasting among HIV positive children in studies conducted by follow up were found to be higher compared to cross-sectional once.  The pooled prevalence of stunting among HIV positive children in East Africa found to be 49.68% (95%CI; 42.59–56.77). This result is higher compared to large scale study conducted on children with HIV positive in Central and West African (33%) HIV care programmes supported by the Growing up Programme in 2011. But this study is lower than the study conducted in southern Africa, which accounts (61.1%) of HIV positive children were chronically under nourished. This difference might be due to the action of non-governmental organizations on providing child nutrition compared to our study area.  The sub group analysis result based on country shows, greater than half (51.63%) of HIV positive children’s in Ethiopia found to be stunted followed by nearly half (48.21%) of HIV positive children’s in Uganda are under this scheme. This result is higher compared the data reported by mini EDHS, 2019 accounted (37%), and large scale DHS study conducted in sub-Saharan countries, which accounted 26.2% in Ethiopia and 38.2% in Rwanda. The inconsistency between results might be due to difference number and geographical area of study participants. This result calls the integration of nutritional support for HIV positive children and early initiation of ART to loosen the burden of chronic under nutrition in East African Countries. | 30 |
| Limitations | 25 | **Strength and limitations**  As strength the authors used a pre-specified protocol for search strategy, data abstraction, and quality assessment. The included studies were low risk of bias based on JBI quality assessment checklist. Moreover, we employed subgroup analysis based on study country, study design, and year of publication and sensitivity analysis to identify the small study effect and the risk of heterogeneity.  Nevertheless, there may be publication bias because not all grey literature are included and language bias; since all included studies are published in English. | 21 |
| Conclusions | 26 | The results presented above provide evidence of a higher prevalence of undernutrition among HIV positive children in East Africa. Despite the country level variations of child undernutrition in East Africa, still it is high in all aspects compared to the studies from other parts of Africa. It is recommended that further systematic review and meta-analysis need to be conducted on magnitude of malnutrition among HIV positive children in Sub-Saharan Africa as a whole. | 21 |
| **FUNDING** | | |  |
| Funding | 27 | N/A |  |

*From:*  Moher D, Liberati A, Tetzlaff J, Altman DG, The PRISMA Group (2009). Preferred Reporting Items for Systematic Reviews and Meta-Analyses: The PRISMA Statement. PLoS Med 6(7): e1000097. doi:10.1371/journal.pmed1000097

For more information, visit: **www.prisma-statement.org**.

Page 2 of 2
