# Supplementary material for: Magnitude of underweight, wasting and stunting among HIV positive children in East Africa: A systematic review and meta-analysis
Source: PLoS One. 2020 Sep 17;15(9):e0238403. doi: 10.1371/journal.pone.0238403 (PMC7498078; doi:10.1371/journal.pone.0238403)
Supplement: S1 Table — (DOCX) [file pone.0238403.s002.docx]

# Table S1. Search strategy used for one of the databases

| Medline/PubMed | | |  |
| --- | --- | --- | --- |
|  | **Search terms** | |  |
| Group | **Non-MeSH terms** | **MeSH (sub-terms in MeSH)** | **Citations** |
| #1 | Prevalence  Magnitude |  |  |
| #2 | Infant  Child | Children |  |
| #3 | underweight  wasting  stunting | Under nutrition |  |
| #4 | Eastern Africa |  |  |
| #1 AND #2 AND #3 AND #4 AND #5 |  |  | **2252** |

(Prevalence OR magnitude) AND (children [MeSH Terms] OR child OR infant) AND (under-nutrition [MeSH Terms] OR underweight OR wasting OR stunting) AND Eastern Africa
