# Supplementary material for: Magnitude of underweight, wasting and stunting among HIV positive children in East Africa: A systematic review and meta-analysis
Source: PLoS One. 2020 Sep 17;15(9):e0238403. doi: 10.1371/journal.pone.0238403 (PMC7498078; doi:10.1371/journal.pone.0238403)
Supplement: S2 Table — Using Joanna Briggs Institute (JBI) quality appraisal checklist [16]. (DOCX) [file pone.0238403.s003.docx]

Table S2: Quality appraisal result of included studies in East Africa, from January 2000-December 2019.; Using Joanna Briggs Institute (JBI) quality appraisal checklist [16],

| **Author** | **Quality assessment questions** | | | | | | | | | | |  |  |  |
| --- | --- | --- | --- | --- | --- | --- | --- | --- | --- | --- | --- | --- | --- | --- |
|  | Q1 | Q2 | Q3 | Q4 | Q5 | Q6 | Q7 | Q8 | Q9 | Q10 | Q11 | Yes Total | Quality status | Overall appraisal |
| Cross-sectional studies | | | | | | | | | | | | | | |
| Mekonen *et al* (1) | N | Y | N | Y | Y | N | Y | Y |  |  |  | 5/8 | Low risk | Included |
| Jeylan *et al* (2) | Y | Y | Y | Y | Y | Y | Y | Y |  |  |  | 8/8 | Low risk | Included |
| Abdulkadir *et al* (3) | Y | Y | UC | Y | Y | N | Y | Y |  |  |  | 6/8 | Low risk | Included |
| Haileselassie *et al* (4) | Y | Y | Y | Y | Y | UC | Y | Y |  |  |  | 7/8 | Low risk | Included |
| Teklemariam *et al* (5) | Y | UC | Y | Y | Y | N | Y | Y |  |  |  | 6/8 | Low risk | Included |
| Wondimu *et al* (6) | Y | N | Y | Y | Y | UC | Y | Y |  |  |  | 6/8 | Low risk | Included |
| Megabiaw *et al* (7) | Y | UC | Y | Y | Y | N | Y | Y |  |  |  | 6/8 | Low risk | Included |
| Arpadi *et al* (8) | Y | UC | Y | Y | Y | N | Y | Y |  |  |  | 6/8 | Low risk | Included |
| Nalwoga *et al* (9) | UC | Y | Y | Y | Y | N | Y | Y |  |  |  | 5/8 | Low risk | Included |
| Sunguya *et al* (10) | Y | Y | Y | Y | Y | UC | Y | Y |  |  |  | 7/8 | Low risk | Included |
| Herman *et al* (11) | Y | Y | Y | Y | Y | N | Y | Y |  |  |  | 7/8 | Low risk | Included |
| Sunguya *et al* (12) | Y | UC | Y | Y | Y | N | Y | Y |  |  |  | 6/8 | Low risk | Included |
| Dundigalla *et al* (13) | Y | Y | Y | Y | Y | Y | Y | Y |  |  |  | 8/8 | Low risk | Included |
| **Cohort studies** | | | | | | | | | | | | | | |
| Kedir *et al* (14) | Y | Y | Y | UC | UC | Y | Y | Y | Y | Y | N | 8/11 | Low risk | Included |
| Tekleab *et al* (15) | Y | Y | Y | Y | UC | Y | Y | Y | Y | Y | Y | 10/11 | Low risk | Included |
| Yassin *et al* (16) | Y | Y | Y | Y | UC | Y | Y | Y | Y | Y | N | 9/11 | Low risk | Included |
| Workneh *et al* (17) | Y | Y | Y | Y | Y | Y | Y | Y | Y | Y | Y | 11/11 | Low risk | Included |
| Kamenju *et al* (18) | Y | Y | Y | Y | UC | Y | Y | Y | Y | Y | Y | 10/11 | Low risk | Included |
| Arinaitwe *et al* (19) | Y | Y | Y | Y | UC | Y | Y | Y | Y | Y | N | 9/11 | Low risk | Included |
| Mwiru *et al* (20) | Y | Y | Y | Y | Y | Y | Y | Y | Y | Y | Y | 11/11 | Low risk | Included |
| Mwiru *et al*(21) | Y | Y | Y | Y | Y | Y | Y | Y | Y | Y | Y | 11/11 | Low risk | Included |

Key: Y=yes, N=no, UC=unclear, Q=Question

**References**

1. Mekonnen A. Assessment of magnitude and factors affecting nutritional status of HIV infected under-five children at five public hospitals in Addis Ababa and its programmatic implication 2014.

2. Jeylan A, Mohammed E, Girma A. Magnitude of Stunting, Thinness and Associated Factors among HIV Positive Children Attending Chronic HIV Care and Support in Adama Hospital Medical College, Adama, Oromia Regional State, Ethiopia.

3. Gondar E. PREVALENCE OF MALNUTRITION AND ASSOCIATED FACTORS AMONG HIV-INFECTED CHILDREN AGED 6-59 MONTHS AT GONDAR UNIVERSITY HOSPITAL, NORTHWEST ETHIOPIA: DEPARTMENT OF PEDIATRICS AND CHILDHEALTH, COLLEGE OF MEDICINE AND HEATLH …; 2014.

4. Haileselassie B, Roba KT, Weldegebreal F. Undernutrition and its Associated Factors among Pediatric Age Children Attending Antiretroviral Therapy in Eastern Ethiopia. East African Journal of Health and Biomedical Sciences. 2019;3(1):1-12.

5. Teklemariam Z, Mitiku H, Mesfin F. Prevalence of anemia and nutritional status among HIV-positive children receiving antiretroviral therapy in Harar, eastern Ethiopa. HIV/AIDS (Auckland, NZ). 2015;7:191.

6. Wondimu WB. Nutritional status and associated factors in human immunodeficiency virus infected children in Hawassa University Referral Hospital Hawassa City. SNNPR, Ethiopia, EPHI. 2014.

7. Megabiaw B, Wassie B, Rogers NL. Malnutrition among HIV-Positive Children at two Referral Hospitals in Northwest Ethiopia. Ethiop J Health Biomed Sci. 2012;5:3-10.

8. Arpadi S, Lamb M, Nzeyimana IN, Vandebriel G, Anyalechi G, Wong M, et al. Better Outcomes Among HIV-Infected Rwandan Children 18–60 Months of Age After the Implementation of “Treat All”. Journal of acquired immune deficiency syndromes (1999). 2019;80(3):e74.

9. Nalwoga A, Maher D, Todd J, Karabarinde A, Biraro S, Grosskurth H. Nutritional status of children living in a community with high HIV prevalence in rural Uganda: a cross‐sectional population‐based survey. Tropical medicine & international health. 2010;15(4):414-22.

10. Sunguya BF, Poudel KC, Mlunde LB, Urassa DP, Yasuoka J, Jimba M. Poor nutrition status and associated feeding practices among HIV-positive children in a food secure region in Tanzania: a call for tailored nutrition training. PloS one. 2014;9(5).

11. Herman ND. HIV and child malnutrition: Examining the relationship of maternal HIV on nutritional outcomes for dependent children in Kenya: Georgetown University; 2012.

12. Sunguya BF, Poudel KC, Otsuka K, Yasuoka J, Mlunde LB, Urassa DP, et al. Undernutrition among HIV-positive children in Dar es Salaam, Tanzania: antiretroviral therapy alone is not enough. BMC public health. 2011;11(1):869.

13. Dundigalla C, Chidugulla SK, Ashwani N, Divya BP, Dundigalla P. Study of Prevalence Of Malnutrition In HIV Positive Children and Its Correlation With Cd4 Count. IOSR J of Denl and Med Sci. 2015;14(12):50-7.

14. Kedir A, Desta A, Fesseha G. Factors affecting survival of HIV positive children taking antiretroviral therapy at Adama Referral Hospital and Medical College, Ethiopia. J AIDS Clin Res. 2014;5(3):1-6.

15. Tekleab AM, Tadesse BT, Giref AZ, Shimelis D, Gebre M. Anthropometric improvement among HIV infected pre-school children following initiation of first line anti-retroviral therapy: implications for follow up. PloS one. 2016;11(12).

16. Yassin S, Gebretekle GB. Magnitude and predictors of antiretroviral treatment failure among HIV‐infected children in Fiche and Kuyu hospitals, Oromia region, Ethiopia: a retrospective cohort study. Pharmacology research & perspectives. 2017;5(1):e00296.

17. Workneh N, Girma T, Woldie M. Immunologic and clinical outcomes of children on HAART: a Retrospective cohort analysis at Jimma University specialized hospital. Ethiopian Journal of Health Sciences. 2009;19(2).

18. Kamenju P, Liu E, Hertzmark E, Spiegelman D, Kisenge R, Kupka R, et al. Nutritional status and complementary feeding among HIV‐exposed infants: a prospective cohort study. Maternal & child nutrition. 2017;13(3):e12358.

19. Arinaitwe E, Gasasira A, Verret W, Homsy J, Wanzira H, Kakuru A, et al. The association between malnutrition and the incidence of malaria among young HIV-infected and-uninfected Ugandan children: a prospective study. Malaria journal. 2012;11(1):90.

20. Mwiru RS, Spiegelman D, Duggan C, Seage III GR, Semu H, Chalamilla G, et al. Nutritional status and other baseline predictors of mortality among HIV-infected children initiating antiretroviral therapy in Tanzania. Journal of the International Association of Providers of AIDS Care (JIAPAC). 2015;14(2):172-9.

21. Mwiru RS, Spiegelman D, Duggan C, Seage III GR, Semu H, Chalamilla G, et al. Growth among HIV-infected children receiving antiretroviral therapy in Dar es Salaam, Tanzania. Journal of tropical pediatrics. 2014;60(3):179-88.
