# Supplementary material for: Magnitude of underweight, wasting and stunting among HIV positive children in East Africa: A systematic review and meta-analysis
Source: PLoS One. 2020 Sep 17;15(9):e0238403. doi: 10.1371/journal.pone.0238403 (PMC7498078; doi:10.1371/journal.pone.0238403)
Supplement: S1 Fig — (DOCX) [file pone.0238403.s004.docx]

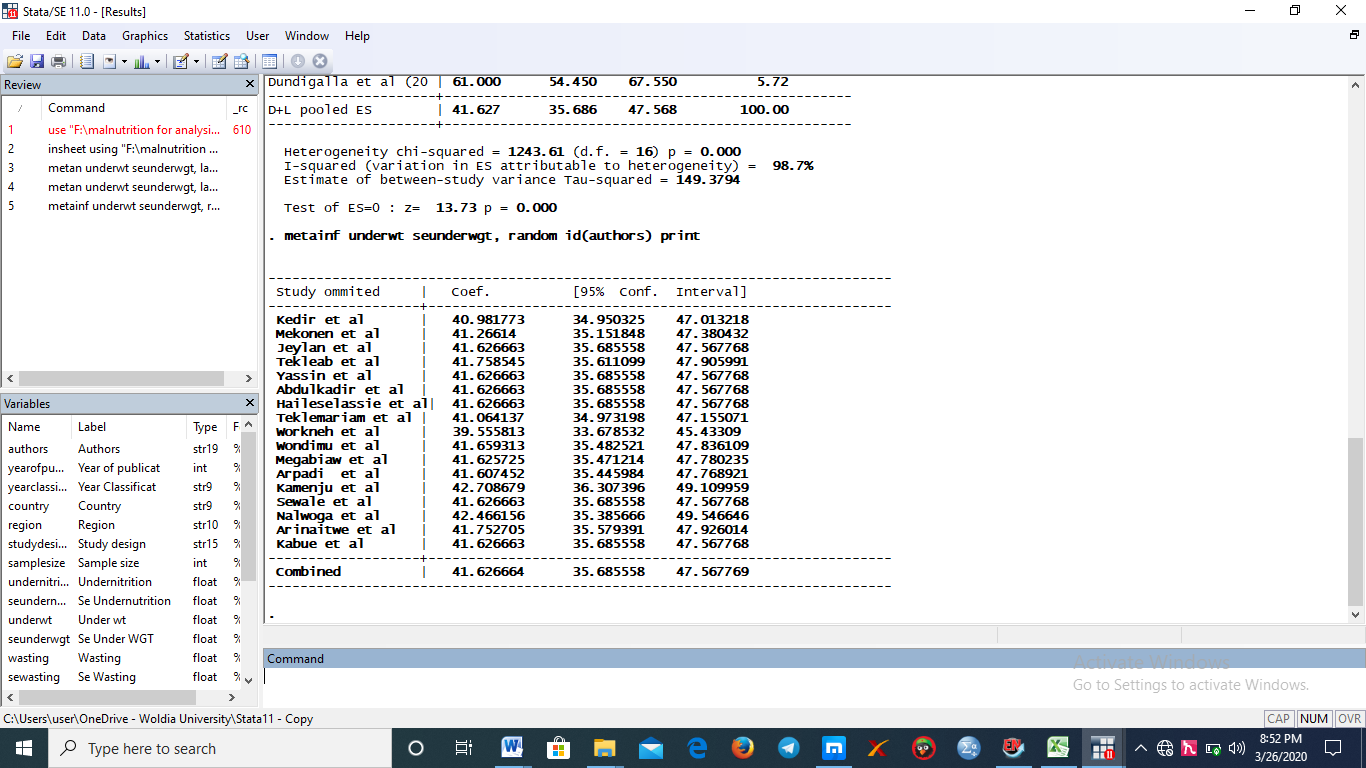


Figure S1: Forest plot showing the sensitivity analysis of the prevalence of under-weight among HIV positive children in East Africa, from January 2008-December 2019
