# Supplementary material for: Magnitude of underweight, wasting and stunting among HIV positive children in East Africa: A systematic review and meta-analysis
Source: PLoS One. 2020 Sep 17;15(9):e0238403. doi: 10.1371/journal.pone.0238403 (PMC7498078; doi:10.1371/journal.pone.0238403)
Supplement: S6 Fig — (DOCX) [file pone.0238403.s009.docx]

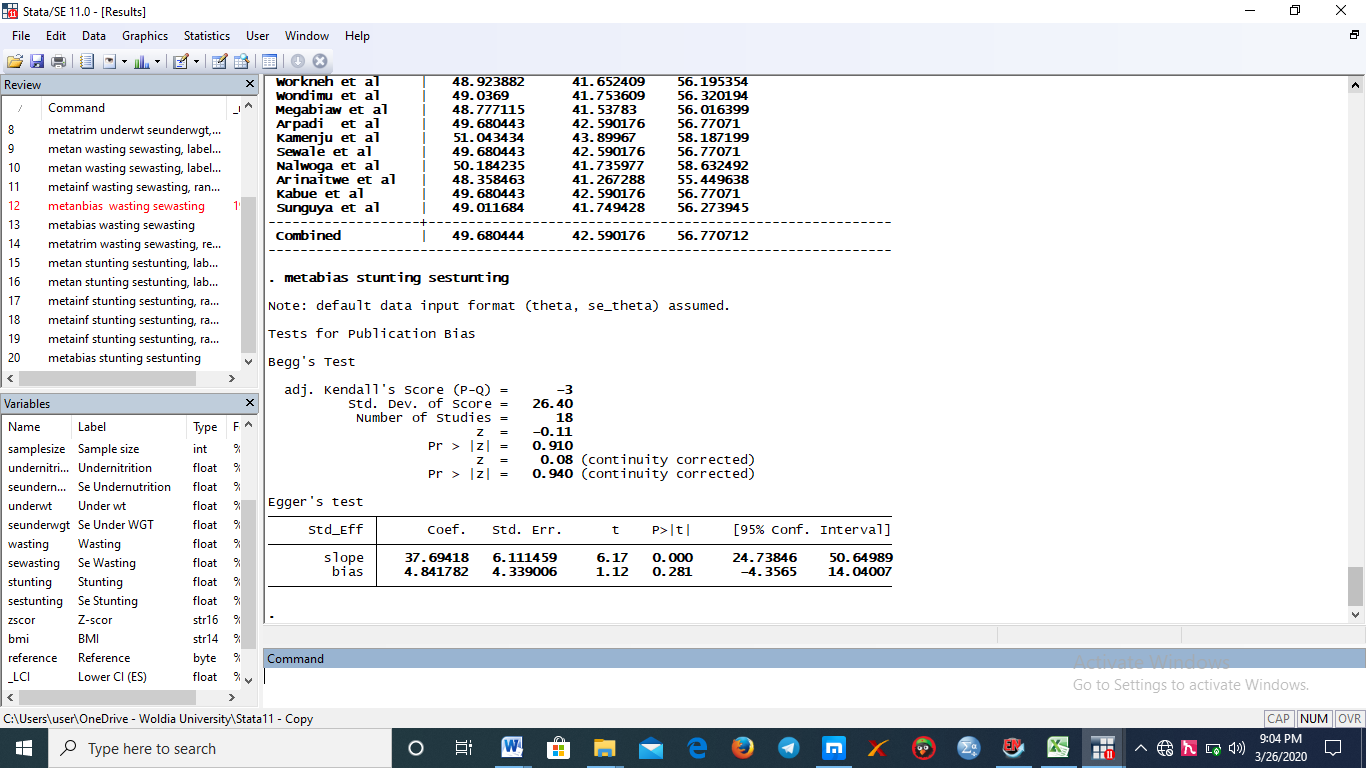


Figure S6: Publication bias the pooled prevalence of stunting in East Africa, from January 2008-December 2019
