# Supplementary material for: Magnitude of underweight, wasting and stunting among HIV positive children in East Africa: A systematic review and meta-analysis
Source: PLoS One. 2020 Sep 17;15(9):e0238403. doi: 10.1371/journal.pone.0238403 (PMC7498078; doi:10.1371/journal.pone.0238403)
Supplement: S1 Dataset — (DOCX) [file pone.0238403.s010.docx]

| Author name | Publication Year | Country | Region | Study design | Sample size |  | Prevalence of under-nutrition, wasting, underweight, stunting | | | Oper def /cut of point | | Reference by endnote |
| --- | --- | --- | --- | --- | --- | --- | --- | --- | --- | --- | --- | --- |
|  |  |  |  |  |  | Under nutrition | Under wt | Wasting | Stunting | Z-scor | BMI |  |
| 1. Kedir eta al | 2014 | Ethiopia | Adama/Et | Cohort | 560 |  | 51.6 |  |  | <-2sd | <-2sd | (1) |
| 1. Mekonen A | 2014 | Ethiopia | AA/ET | Cross sectional | 255 |  | 47.5 |  | 71.3 |  |  | (2) |
| 1. Jeylan et.al. | 2018 | Ethiopia | Adama/ET | Cross sectional | 412 |  |  | 21.8 | 13.4 | <-2sd |  | (3) |
| 1. Tekleab et al | 2016 | Ethiopia | AA/ET | Cohort | 202 |  | 39.5 | 16.3 | 71.3 | <-2sd |  | (4) |
| 1. Yassin et al |  | Ethiopia | Fiche/ET | Cohort | 269 | 44.71 |  |  |  | <3rd centile |  | (5) |
| 1. Abdulkadir et al |  | Ethiopia | Gonder/ET | Cross sectional | 142 | 68.3 |  | 31.7 | 46.5 | NR | NR |  |
| 1. Haileselassie et al. | 2019 | Ethiopia | Harar/Et | Cross sectional | 390 |  |  | 28.2 | 24.7 | <-2sd |  |  |
| 1. Teklemariam et al | 2015 | Ethiopia | Harar/ET | Cross sectional | 108 |  | 51.6 | 31.5 | 49.1 | <-2sd | <-2sd(w |  |
| 1. Netsanet W. et al | 2009 | Ethiopia | Jimma/ET | Cohort | 96 |  | 77.1 | 47.5 | 63.5 | <5^th^ centile | <90%(w |  |
| 1. Wondimu WB | 2014 | Ethiopia | Hawassa/Et | Cross sectional | 455 |  | 41.2 | 21.4 | 60.5 | <-2sd | WHZ <-2sd |  |
| 1. Megabiaw et al | 2012 | Ethiopia | Gondar/ET | Cross sectional | 301 |  | 41.7 | 5.8 | 65 | <-2sd |  |  |
| 1. Arpadi et al | 2019 | Rwanda | Rwanda | Cross sectional | 374 |  | 42 |  |  | <-2sd |  |  |
| 1. Kamenju et al | 2017 | Tanzania | Tanzania | Cohort | 2092 |  | 25.4 | 21.6 | 27.1 | <-2sd |  |  |
| 1. **Sewale et al.** | 2018 | Ethiopia | Gojjam/Et | Cross sectional | 372 | 60.2 |  |  |  | <-2 sd |  |  |
| 1. Nalwoga et al | 2010 | Uganda | Uganda | Cross sectional | 5951 |  | 30 | 10 | 42 | <-2 sd | <-2 sd |  |
| 1. Arinaitwe et al. | 2012 | Uganda | Uganda | Cohort | 358/259 exp |  | 39.7 |  | 71.8 | <-2 sd m-s | -1-(-2) |  |
| 1. Kabue et al | 2008 | Uganda | Uganda | Cohort | 749 |  |  |  |  | <5^th^centile(udwt | <5^th^ centi(stu |  |
| 1. Sunguya BF et al | 2014 | Tanzania | Tanzania | Cross sectional | 748 |  | 40.6 | 30.2 | 60.8 | <-2sd | <-2sd |  |
| 1. Herman et al | 2102 | Kenya | Kenya | Cross sectional | 2275 |  | 19.4 | 7 | 28.6 | <-2 sd | <-2 sd |  |
| 1. Mwiru et al | 2015 | Tanzania | Tanzania | Cohort | 3144 |  | 53 | 33 | 56 | <-2 sd | <-2 sd |  |
|  |  |  |  |  |  |  |  |  |  |  |  |  |
|  |  |  |  |  |  |  |  |  |  |  |  |  |
|  |  |  |  |  |  |  |  |  |  |  |  |  |
|  |  |  |  |  |  |  |  |  |  |  |  |  |

1. Kedir A, Desta A, Fesseha G. Factors affecting survival of HIV positive children taking antiretroviral therapy at Adama Referral Hospital and Medical College, Ethiopia. J AIDS Clin Res. 2014;5(3):1-6.

2. Mekonnen A. Assessment of magnitude and factors affecting nutritional status of HIV infected under-five children at five public hospitals in Addis Ababa and its programmatic implication 2014.

3. Jeylan A, Mohammed E, Girma A. Magnitude of Stunting, Thinness and Associated Factors among HIV Positive Children Attending Chronic HIV Care and Support in Adama Hospital Medical College, Adama, Oromia Regional State, Ethiopia.

4. Tekleab AM, Tadesse BT, Giref AZ, Shimelis D, Gebre M. Anthropometric improvement among HIV infected pre-school children following initiation of first line anti-retroviral therapy: implications for follow up. PloS one. 2016;11(12).

5. Yassin S, Gebretekle GB. Magnitude and predictors of antiretroviral treatment failure among HIV‐infected children in Fiche and Kuyu hospitals, Oromia region, Ethiopia: a retrospective cohort study. Pharmacology research & perspectives. 2017;5(1):e00296.
